# Decoding Host–Pathogen Dynamics in *Klebsiella pneumoniae* Infections: Mechanisms of Cell Death Regulation and Host-Directed Therapies for Sepsis

**DOI:** 10.3390/antibiotics15050505

**Published:** 2026-05-18

**Authors:** Shwetha Susan Thomas, Krishnakripa Kannan, Kuniyil Abhinand, Arjun M. Menon, Gayathri Ranjith, Shima Merin Sony, Pradeesh Babu, Geetha B. Kumar, Bipin G. Nair, KB Arun, Lekshmi K. Edison, Aravind Madhavan

**Affiliations:** 1 School of Biotechnology, Amrita Vishwa Vidyapeetham, Amritapuri, Kollam 690525, Kerala, India; 2 Department of Life Sciences, CHRIST, Deemed to be University, Bangalore 560029, Karnataka, India; 3 Department of Comparative, Diagnostic, and Population Medicine, College of Veterinary Medicine, University of Florida, Gainesville, FL 32610, USA

**Keywords:** *Klebsiella pneumoniae*, innate immunity, cell death, sepsis, host–pathogen interactions, host-directed therapy

## Abstract

Pathogenic microbes utilize virulence strategies to subvert host immune responses, highlighting a continuous race between the pathogen and the host. The emergence of multidrug- resistant and hypervirulent strains of *Klebsiella pneumoniae* (*Kp*) poses a critical threat to public health. This critical evaluation identifies key gaps in understanding the interplay between *Kp* and host innate immunity. This review provides a comprehensive overview of the mechanisms by which *Kp* triggers various forms of cell death. Dysregulated cell death may exacerbate cytokine release, contributing to the hyperinflammatory response characteristic of sepsis. The rise in antimicrobial resistance (AMR) in *Kp* necessitates the exploration of alternative therapeutic approaches. This review highlights that immunomodulatory approaches targeting cell death regulators or immune checkpoints may offer host-directed strategies against *Kp*-induced sepsis. Although novel immunotherapies offer potential to restore immune balance, their clinical applicability remains constrained by limited translational evidence.

## 1. Introduction

*Klebsiella pneumoniae* (*Kp*) is a Gram-negative opportunistic pathogen and a major global public health threat, particularly in healthcare settings, where it is a leading cause of hospital-acquired infections among immunocompromised individuals. It accounts for approximately 11.8% of hospital-acquired pneumonia cases and 8–12% of ventilator-associated pneumonia, compared to ~7% in non-ventilated patients [1]. In addition, *Kp* is a frequent cause of urinary tract infections and bacteremia, with mortality rates reaching 50–100% in individuals with underlying conditions such as alcoholism and septicemia [1]. In India, surveillance data from the Indian Council of Medical Research (ICMR) indicate a concerning decline in antibiotic susceptibility, with piperacillin–tazobactam susceptibility decreasing from 42.6% to 26.5%, imipenem from 58.5% to 35.6%, meropenem from 48% to 37.6%, and ciprofloxacin from 32% to 17.1% [2]. These trends underscore the urgent threat posed by multidrug-resistant (MDR) and carbapenem-resistant *Kp* (CR *Kp*).

A critical determinant of *Kp* pathogenesis is its ability to manipulate host cell death pathways—including pyroptosis, apoptosis, and necroptosis to facilitate immune evasion, intracellular persistence, and disease progression. Pyroptosis is a highly inflammatory form of programmed cell death mediated by gasdermins and characterized by inflammasome activation, caspase-1 cleavage, and the release of pro-inflammatory cytokines such as IL-1β and IL-18 [3]. *K. pneumoniae* induces pyroptosis in alveolar macrophages through activation of the NLRP3 inflammasome, ASC recruitment, and gasdermin D (GSDMD) cleavage, with hypervirulent strains (hvKP) eliciting more robust inflammasome activation than classical strains [4]. Moreover, antibiotic-induced outer membrane vesicles (OMVs) derived from *Kp* promote caspase-11–dependent non-canonical pyroptosis, exacerbating lung inflammation and tissue injury [5]. The pathogen exploits excessive pyroptosis to drive neutrophil recruitment and cytokine release while evading phagocytic clearance through virulence determinants. Thus, *Kp* finely balances inflammasome activation to promote inflammation without effective bacterial elimination, contributing to chronic infection [4,6].

In parallel, *Kp* induces apoptosis, a caspase-dependent and generally non-inflammatory form of programmed cell death, further enhancing immune evasion and virulence [7]. The bacteriocin microcin E492 triggers apoptosis through calcium dysregulation and caspase activation, which will be discussed later in the review [8]. Infection-associated inflammation and immune dysregulation further exacerbate apoptotic cell death, impairing efferocytosis and facilitating bacterial persistence and systemic dissemination [9,10]. Beyond pyroptosis and apoptosis, necroptosis represents a caspase-independent, inflammatory form of programmed necrosis driven by RIPK and MLKL proteins, culminating in membrane rupture and release of damage-associated molecular patterns (DAMPs) [11]. *Kp* exploits necroptosis through virulence factors such as lipopolysaccharide (LPS) and pore-forming toxins, and enhances bacterial dissemination [12].

Sepsis represents a severe and life-threatening consequence of dysregulated host responses to infection, characterized by widespread inflammation, tissue damage, and multi-organ failure. According to the World Health Organization (WHO), approximately 48.9 million cases of sepsis occurred globally in 2017, resulting in nearly 11 million deaths—accounting for ~20% of all global mortality—with around 40% of cases affecting children under five years of age [13,14]. A central feature of sepsis is the cytokine storm, driven by excessive release of inflammatory mediators such as IL-6, IL-1β, TNF-α, and interferons. Pattern recognition receptors, including Toll-like receptors (particularly TLR4), and inflammasomes such as NLRP3, detect pathogen-associated molecular patterns (PAMPs) and damage-associated molecular patterns (DAMPs), triggering uncontrolled inflammatory cascades [15,16,17]. This hyperactivation leads to endothelial dysfunction, vascular leakage, immune paralysis, and exacerbation of sepsis pathology [18].

Hypervirulent *Kp* strains further amplify septic inflammation by activating NF-κB and STAT1 signaling pathways, promoting excessive M1 macrophage polarization and neutrophil hyperactivation, which collectively contribute to rapid disease progression and high mortality [19]. Despite advances in antimicrobial therapy, the rising prevalence of hv *Kp* and CR *Kp* strains severely limits treatment options and underscores the need for alternative therapeutic approaches. Addressing *Kp* infections, therefore, necessitates the development of innovative strategies beyond conventional antibiotics. Immunotherapeutic and host-directed approaches targeting bacterial immune evasion mechanisms represent promising alternatives. However, a critical knowledge gap remains in understanding how *Kp* dynamically modulates host innate immune responses.

This review critically examines current insights into *Kp*–induced immune modulation, with a specific focus on the pathogen’s ability to manipulate host cell death signaling pathways to enhance virulence and pathogenesis. A comprehensive search strategy was employed to cover the relevant literature in the field. The PubMed platform was used to collect information with search terms included mainly ‘*Klebsiella pneumoniae*’, ‘sepsis’, ‘pyroptosis’, ‘apoptosis’, ‘necroptosis’, ‘immune evasion’, etc. Articles published between 2015 and 2026 were considered for the process. Studies were selected based on relevance to mechanistic insights and immunological outcomes, with priority given to recent and impactful findings. By elucidating these underexplored host–pathogen interactions, this work aims to provide a mechanistic framework for the rational design of immunotherapeutic and host-directed interventions. Given the increasing global burden of *Kp*, uncovering the molecular basis of its immune evasion strategies is both timely and essential for the development of effective, next-generation therapeutic approaches.

## 2. Immune Evasion Strategies Employed by *Klebsiella pneumoniae*

*Kp* deploys a multifaceted arsenal of immune evasion strategies that enable survival, persistence, and dissemination within the host. Central to these strategies is the capsular polysaccharide (CPS), a major virulence determinant that masks pathogen-associated molecular patterns (PAMPs), including lipopolysaccharide (LPS), from host pattern recognition receptors (PRRs). By shielding LPS from Toll-like receptor 4 (TLR4) recognition, the capsule dampens innate immune activation and inflammatory signaling [20]. In parallel, CPS interferes with complement activation by inhibiting C3b deposition and membrane attack complex (MAC) formation, thereby blocking opsonophagocytosis and promoting bacterial survival in serum and tissues [21].

*Kp* further enhances immune evasion through capsular switching and structural remodeling of its polysaccharides. Alterations in capsule glycan composition impair recognition by the lectin pathway of complement activation, while sialylation of the capsule inhibits complement-mediated phagocytosis. Enzymatic removal of sialic acid residues restores phagocytic susceptibility, underscoring the functional importance of capsule sialylation in immune evasion [22,23]. In addition to the capsule, *Kp* modifies its LPS to evade host defenses. The capsule masks LPS from immune detection, whereas elongation of the O-antigen in smooth LPS confers increased resistance to complement-mediated killing. In contrast, rough LPS mutants lacking O-antigens exhibit heightened susceptibility to serum bactericidal activity [22].

Structural modifications of lipid A further contribute to immune evasion and antimicrobial resistance. *Kp* alters lipid A through LpxO-mediated hydroxylation of the R-3-hydroxymyristoyl group, a process regulated by the PhoPQ two-component system. These 2-hydroxylated hexa-acylated lipid A species enhance outer membrane stability, reduce recognition by innate immune receptors, and increase resistance to host-derived antimicrobial peptides (AMPs), thereby facilitating infection persistence [24]. Outer membrane protein A (OmpA) is also implicated in adaptation to AMP-mediated stress, potentially through regulation of the σ^E^ envelope stress response pathway [25]. Notably, exposure to polymyxins induces cross-resistance to both polymyxin antibiotics and host AMPs by upregulating capsule biosynthesis genes and lipid A modification pathways, reinforcing bacterial defense mechanisms under immune pressure [26].

*Kp* also subverts innate immune clearance by interfering with neutrophil turnover and efferocytosis. The pathogen suppresses phosphatidylserine exposure on dying neutrophils through enhanced flippase activity and biases cell death toward necroptosis rather than apoptosis. This impairs efferocytosis, leading to neutrophil accumulation, secondary necrosis, and the release of pro-inflammatory cytokines and tissue-damaging enzymes. Pharmacological or genetic targeting of flippases or necroptosis pathways restores efferocytosis and improves disease outcomes in murine pneumonia models, highlighting these pathways as potential therapeutic targets [10,21]. The resulting sustained inflammatory milieu promotes tissue damage and creates a niche favorable for bacterial persistence.

Within macrophages, *Kp* manipulates intracellular trafficking to avoid lysosomal degradation. The bacterium exploits the PI3K–Akt–Rab14 signaling axis to prevent fusion of the Klebsiella-containing vacuole (KCV) with lysosomes, enabling intracellular survival. Interestingly, capsule expression is dispensable for intracellular persistence in the absence of opsonization, and conditions within the KCV downregulate capsule biosynthesis, suggesting dynamic regulation of virulence traits during infection [27].

*K. pneumoniae* further modulates macrophage polarization to suppress antibacterial immunity. While classically activated (M1) macrophages are critical for intracellular pathogen clearance, excessive activation can cause tissue damage. *Kp* skews macrophage differentiation toward an alternatively activated (M2-like) phenotype, limiting bactericidal functions, and promoting persistence. Experimental enhancement of M1 polarization or depletion of M2 macrophages significantly improves bacterial clearance, underscoring the importance of macrophage plasticity in disease outcome [28,29].

In addition, *Kp* exploits immunosuppressive myeloid populations to dampen host responses. IL-10–producing Gr1^int^ myeloid-derived suppressor cell (MDSC)-like populations expand during infection and suppress neutrophil recruitment and inflammatory resolution. While early IL-10 production impairs bacterial clearance, its complete absence results in excessive neutrophilic inflammation and severe lung injury. During later stages of infection, expansion of these MDSC-like cells promotes efferocytosis of apoptotic neutrophils and attenuates inflammation, facilitating immune evasion and chronic infection [30].

Beyond innate immune modulation, *Kp* interferes with adaptive immunity through secretion system–mediated immune suppression. The type VI secretion system (T6SS) activates the cGAS–STING signaling pathway in host cells, leading to upregulation of the immune checkpoint molecule PD-L1 on interstitial macrophages. This suppresses T cell activation and effector function, promoting immune exhaustion and bacterial persistence. In STING-deficient models, bacterial targeting shifts toward alveolar macrophages, enhancing bacterial clearance, and highlighting the role of the T6SS–cGAS–STING–PD-L1 axis in immune evasion [31,32].

**Figure 1:**
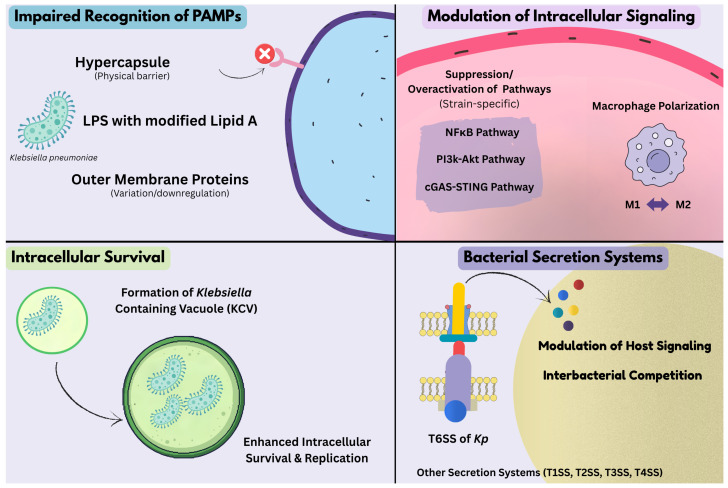
Immune Evasion Strategies by *Kp*. *Kp* employs coordinated mechanisms to modulate host immune responses. At the level of pathogen recognition, the structural modifications in capsule, LPS, can impair the recognition by receptors. At the intracellular level, it could modulate various host signaling pathways. These changes are also associated with shifts in macrophage polarization. At the effector stage, it can persist within vacuoles and enhance survival. Bacterial secretion systems also contribute to immune modulation, ultimately providing survival and pathogenesis.

Collectively, Figure 1 represents these diverse and interconnected immune evasion strategies by *Kp* to manipulate host innate and adaptive immune responses, promote immune dysregulation, and establish persistent infections. Beyond pathogen-driven mechanisms, the outcome of *Kp* infection is also profoundly shaped by host genetic factors that influence immune responsiveness, susceptibility, and disease progression. These host determinants form a critical but underexplored dimension of host–pathogen interactions and are discussed in the following section.

## 3. Host Genetic Determinants of Susceptibility to Pneumonia and Sepsis

Host genetic variation plays a critical role in determining susceptibility to pneumonia and sepsis, as well as disease severity and clinical outcomes. Polymorphisms in genes governing innate and adaptive immune responses influence pathogen recognition, inflammatory signaling, and immune regulation, thereby shaping host–pathogen interactions during infection. Genetic variation within the major histocompatibility complex (MHC) locus, which encodes human leukocyte antigen (HLA) molecules, is central to antigen presentation and adaptive immune activation. Specific HLA alleles modulate peptide binding and T-cell recognition, influencing pathogen clearance and immunopathology. However, the high degree of polymorphism and dense gene clustering within the MHC region complicate genetic association studies and limit definitive attribution of disease risk to individual alleles [33].

Polymorphisms in pattern recognition receptors, particularly Toll-like receptors (TLRs), further contribute to interindividual variability in susceptibility to Gram-negative infections and sepsis. The *Asp299Gly* mutation and Thr399Ile mutation in the *TLR4* receptor reveal significant inferences on the same [34,35,36]. Cytokine gene polymorphisms also exert a substantial influence on disease severity by modulating the magnitude and duration of inflammatory responses. The *G1082A* single-nucleotide polymorphism (SNP) in the *IL-10* promoter region has been linked to severe pneumonia [37]. The high-expression TAC haplotype has been linked to an elevated risk of sepsis initiation but may confer partial protection against progression to severe disease, whereas the low-expression CTT haplotype reduces sepsis susceptibility but offers limited protection once severe infection is established [38]. These findings highlight the dual and context-dependent roles of *IFN-γ* in antimicrobial defense and inflammatory pathology. Tumor necrosis factor-α (*TNF-α*) polymorphisms [39] and *IL-4*, a key Th2 cytokine, polymorphisms [40] represent another critical determinant of sepsis severity. The key genetic variations in host genes and their associated clinical outcomes have been listed in Table 1.

Host genetic predisposition, often influenced by ethnic background, can also affect susceptibility to specific *Kp* lineages. In regional epidemiological studies, non-diabetic Chinese populations exhibited a higher prevalence of infections caused by K1 *Kp* strains, whereas diabetic individuals from non-Chinese ethnic groups (including Malays, Indians, and Caucasians) were more frequently infected with non-K1 strains [41]. These observations suggest that host metabolic status and genetic background may interact with bacterial virulence determinants to shape strain-specific infection patterns.

Insights from model organisms further reinforce the concept that host defense mechanisms are genetically programmed and extend beyond pathogen uptake. Studies in *Dictyostelium discoideum* and *Drosophila melanogaster* have identified genes such as *Phg1* and *Kil1* as essential for intracellular bacterial killing, independent of phagocytosis efficiency [42].

Collectively, these studies demonstrate that host genetic polymorphisms not only dictate susceptibility to *Kp* infection but are also likely to influence the downstream cell death pathways as well as the trajectory of sepsis. These variations could critically affect immune sensing, inflammatory regulation, and cytokine signaling, leading to different death modalities. It can also influence the fate of the process, ultimately leading to dysregulated systemic inflammation. Subsequent sections discuss how these genetically driven changes could intersect with cell death pathways, thereby influencing sepsis severity. Understanding how host genetic variation intersects with *Kp* virulence strategies is essential for identifying at-risk populations and for the development of precision immunomodulatory and host-directed therapeutic approaches.

## 4. Impact of *Klebsiella pneumoniae* on Host Cell Metabolism

*Kp*, particularly globally disseminated high-risk clones such as ST258, profoundly reprograms host cellular metabolism to support bacterial survival, persistence, and disease progression. During infection, this induces a metabolic shift in host immune cells away from glucose-dependent oxidative metabolism toward enhanced glutaminolysis and fatty acid oxidation (FAO). Increased reliance on mitochondrial oxidative phosphorylation (OXPHOS) and FAO results in elevated production of reactive oxygen species (ROS) [43]. While excessive ROS is detrimental to host immune cells, *Kp* ST258 displays enhanced resistance to oxidative stress, in part through upregulation of the T6SS, which contributes to bacterial fitness and survival in ROS-rich environments [43,44]. Concomitantly, *Kp* infection promotes the accumulation and metabolic programming of immunosuppressive cell populations, including alternatively activated (M2-like) macrophages and myeloid-derived suppressor cells (MDSCs). These cells exhibit altered metabolic profiles characterized by increased FAO and glutamine utilization, which dampen pro-inflammatory and bactericidal functions, thereby facilitating immune evasion and chronic infection [44].

In parallel, infection-associated metabolic remodeling marked by dysregulated glycolysis, increased lactate production, and suppression of effective mitochondrial respiration further compromises antimicrobial effector functions such as phagocytosis, antigen presentation, and cytokine production. *Kp*–induced metabolic rewiring also alters central carbon metabolism. Elevated levels of tricarboxylic acid (TCA) cycle intermediates and disrupted lipid, glutamine, and arginine metabolism collectively increase the availability of key nutrients that support bacterial proliferation and intracellular persistence [45]. Notably, upregulation of glycolysis plays a context-dependent role: while glycolytic flux is essential for mounting effective innate immune responses, excessive or dysregulated glycolysis may inadvertently supply metabolites that *Kp* exploits as carbon and energy sources [46]. This highlights the fine balance between host-protective metabolic activation and pathogen-driven metabolic subversion.

Beyond intracellular metabolism, *Kp* also exploits host membrane lipid composition to enhance virulence. The bacterium interacts with cholesterol-rich membrane lipid rafts, which influence phagocytic uptake and anti-phagocytic properties mediated by the capsular polysaccharide (CPS) [47]. Disruption of lipid raft integrity alters CPS gene regulation and enhances macrophage-mediated bacterial clearance, underscoring the importance of host lipid metabolism in *Kp* pathogenesis [47].

Recognition of these metabolic dependencies has opened new avenues for host-directed therapeutic interventions. Targeting glutaminolysis using glutaminase inhibitors may disrupt the immunosuppressive metabolic landscape that favors bacterial persistence [48]. Similarly, inhibition of fatty acid oxidation has the potential to reprogram immune cell metabolism toward a more bactericidal phenotype while restricting nutrient availability for the pathogen [47].

This metabolic reprogramming should not be considered as a mere consequence but rather a determinant of cell fate and immune function. Shifts in glycolysis, mitochondrial function, or lipid metabolism are known to directly regulate key nodes of programmed cell death. These metabolic shifts induced by *Kp* could drive pathological inflammation, thereby contributing to the progression of sepsis. These insights provide a framework for the subsequent sections, which examine how *Kp* -induced metabolic rewiring interacts with cell death pathways to influence host responses and sepsis progression.

## 5. Comparative Analysis of Cell Death Pathways Induced by *Klebsiella pneumoniae* Infection

**Figure 2:**
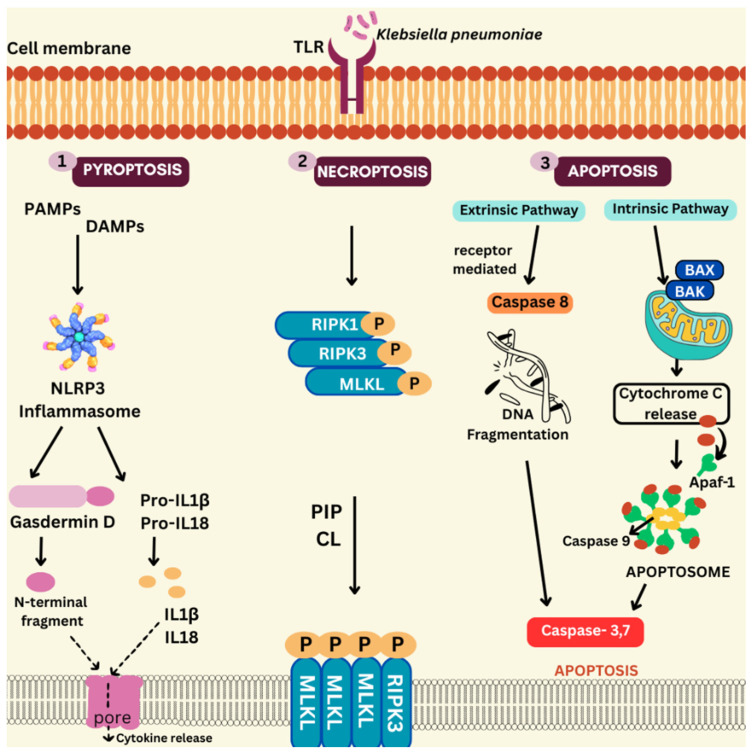
*Kp* infections and their manipulation of cell death pathways. *Kp* modulates key host cell death pathways to regulate inflammation and survival. Pyroptosis is driven by inflammasome activation, caspase-1-mediated gasdermin cleavage, and release of cytokines. Apoptosis is controlled through caspase-8 and mitochondrial pathways. Necroptosis mediated by RIPK and MLKL proteins. PIP: Phosphatidylinositol Phosphate; CL: Cardiolipin.

Cell death is a critical mechanism executed by the hosts as part of the immune defense. This section discusses the various strategies employed by the notorious *Kp* to counteract these cell death pathways. Figure 2 schematically represents the three programmed cell death pathways discussed further.

### 5.1. Pyroptosis

Pyroptosis is a highly inflammatory form of programmed cell death mediated by gasdermin pore formation, characterized by cellular swelling, membrane rupture, and release of cytosolic contents that amplify innate immune responses during microbial infection [4]. A defining feature of pyroptosis is inflammasome activation, leading to caspase-1 cleavage and subsequent maturation and secretion of pro-inflammatory cytokines, including IL-1β, IL-18 [49].

Accumulating evidence demonstrates that *Kp* robustly induces pyroptosis or closely related pyronecrotic cell death in innate immune cells, particularly macrophages. *Kp* infection upregulates core pyroptotic machinery, including NLRP3, cleaved caspase-1, and the N-terminal fragment of gasdermin D (GSDMD-N). Notably, hv *Kp* strains induce significantly higher levels of inflammasome activation and pyroptotic signaling compared with classical KP (cKP), indicating strain-dependent differences in host cell death modulation [3].

In alveolar macrophages, *Kp* -induced pyroptosis is closely linked to transcriptional and post-transcriptional host regulatory mechanisms. Infection upregulates circular RNA circCDC42, which encodes a truncated protein variant (CDC42-165aa) that competitively inhibits DOCK8, suppressing CDC42 GTPase activity. This inhibition triggers Pyrin inflammasome activation, resulting in caspase-1 and GSDMD cleavage, IL-1β release, and pyroptotic cell death. The ensuing exaggerated inflammatory response exacerbates lung injury and contributes to increased mortality during *Kp* infection [50].

Antibiotic exposure further modulates *Kp* -induced pyroptosis. Treatment with carbapenems, particularly imipenem, enhances the production of *Kp* -derived outer membrane vesicles (OMVs) and increases surface exposure of the chaperonin GroEL. GroEL binds to the lectin-like oxidized LDL receptor-1 (LOX-1) on macrophages, promoting OMV internalization and triggering caspase-11-dependent non-canonical pyroptosis through intracellular LPS sensing. This pathway culminates in GSDMD cleavage, membrane rupture, and robust cytokine release. Hv*Kp* strains further exacerbate this response by resisting phagocytosis, resulting in increased tissue damage and heightened expression of NLRP3, ASC, caspase-1, and GSDMD [3].

Capsular components also play a direct role in inflammasome activation. The K1 capsular polysaccharide (K1-CPS) activates the NLRP3 inflammasome in macrophages, inducing IL-1β secretion and pyroptosis [6]. In murine J774A.1 macrophages, K1-CPS, particularly in the presence of ATP, induces caspase-1 activation and IL-1β release, which is markedly reduced following NLRP3 or ASC knockdown. *TLR4* silencing diminishes K1-CPS-induced NLRP3 expression, indicating that TLR4 signaling acts upstream of inflammasome activation. This process is further driven by mitochondrial dysfunction, reactive oxygen species (ROS) generation, and activation of ERK1/2-, JNK1/2-, and p38 MAPK-dependent signaling pathways [6,51].

While pyroptosis is generally considered a host-protective mechanism, its contribution to *Kp* clearance is context-dependent. Effective bacterial elimination relies on the efferocytosis of pyroptotic cells by neighboring phagocytes, which limits secondary necrosis and resolves inflammation. However, *Kp* can exploit excessive or dysregulated pyroptosis to reshape the inflammatory milieu, promoting tissue damage and facilitating bacterial persistence and dissemination [52].

Outer membrane protein A (OmpA) further highlights the dual role of pyroptosis in *Kp* infection. Transcriptomic analyses reveal significant upregulation of pyroptosis-associated genes in OmpA-treated cells compared with whole-bacterial infection. Additionally, OmpA-containing OMVs disrupt cell-cycle progression and enhance ROS production, further amplifying inflammasome activation and pyroptotic signaling [53].

Collectively, these findings demonstrate that *Kp* induces pyroptosis through multiple, overlapping mechanisms involving capsular components, OMVs, antibiotic-induced stress responses, and host regulatory RNAs. While controlled pyroptosis contributes to antimicrobial defense, excessive or misregulated inflammasome activation promotes lung pathology, immune dysregulation, and disease severity. The ability of *Kp,* particularly hvKP and MDR strains, to fine-tune pyroptotic signaling underscores pyroptosis as both a critical host defense mechanism and a vulnerability exploited during infection.

### 5.2. Apoptosis

Apoptosis is a tightly regulated, caspase-dependent form of programmed cell death that plays a critical role in host defense during microbial infections. Its induction, however, is highly context dependent and varies with the pathogen, host cell type, and stage of infection. Pathogens may exploit apoptotic pathways either by inducing apoptosis to damage host tissues and facilitate dissemination or by suppressing apoptosis in immune cells to evade clearance, reflecting diverse strategies of immune modulation [7].

*Kp* employs multiple virulence factors to manipulate apoptotic signaling. The *Kp* -derived bacteriocin microcin E492, a channel-forming peptide, induces apoptosis in human cell lines (HeLa, Jurkat, and RJ2.25). This process is characterized by cell shrinkage, DNA fragmentation, mitochondrial membrane potential loss, and caspase activation, and is effectively blocked by the pan-caspase inhibitor zVAD-fmk. Apoptosis induction is associated with calcium release from intracellular stores, likely resulting from microcin-induced pore formation in cellular membranes [8].

*Kp* infection also induces apoptosis in lung epithelial cells, including HLF-1 and BEAS-2B cell lines, leading to a marked reduction in cell viability. This apoptotic response is driven by mitochondrial dysfunction and upregulation of caspase-9, indicating activation of the intrinsic apoptotic pathway. Epithelial apoptosis compromises barrier integrity, thereby facilitating bacterial invasion and dissemination. Concurrently, *Kp* infection triggers robust inflammatory responses characterized by elevated expression of IL-6, CXCL1, and CXCL2, which exacerbate lung injury and further promote disease progression [54].

Host-derived modulators can counteract *Kp* -induced apoptosis. Clara cell secretory protein 16 (CC16) significantly attenuates *Kp* -induced epithelial apoptosis by suppressing caspase-3/7 activity and reducing oxidative stress. *Kp* infection promotes excessive ROS accumulation, disrupting redox homeostasis and triggering apoptotic pathways. CC16 overexpression inhibits both cytosolic and mitochondrial ROS generation and suppresses NF-κB activation, resulting in reduced production of pro-inflammatory cytokines such as IL-1β, IL-6, and IL-8 [55]. These findings highlight CC16 as a promising host-directed therapeutic candidate capable of mitigating both apoptosis and inflammation.

Importantly, *Kp* regulates apoptosis in a cell-type–specific manner to optimize survival and virulence. While apoptosis is induced in epithelial cells, hepatocytes, and platelets to promote tissue damage and bacterial dissemination, apoptosis is often suppressed in macrophages and neutrophils to prolong intracellular survival and evade immune clearance [3,27]. In murine models, quantitative PCR and immunohistochemical analyses reveal increased caspase-3 expression and a reduced Bcl-2/Bax ratio, indicating enhanced apoptotic signaling in lung tissues [3].

Hypermucoviscous *Kp* (hm*Kp*) strains are especially adept at modulating apoptosis. Although hm*Kp* is associated with increased apoptosis in epithelial and hepatic cells, strain-specific differences exist. In macrophages, apoptosis is often suppressed to enable intracellular persistence, while defective efferocytosis further impairs the clearance of apoptotic cells, weakening host immunity [6]. Similarly, the hv*Kp* -M1 strain inhibits neutrophil apoptosis by upregulating the anti-apoptotic protein Mcl-1, reducing the Bax/Bcl-2 ratio, and blocking caspase-3 activation. Flow cytometry and immunoblotting analyses demonstrate >40% neutrophil survival at 24 h post-infection, coupled with impaired efferocytosis, which delays resolution of inflammation [56].

CPS also contributes to apoptosis induction in lung epithelial cells. CPS-mediated apoptosis requires live bacteria but is independent of specific serotypes. CPS-induced epithelial apoptosis facilitates colonization and tissue invasion [57]. *Kp* -derived OMVs are potent inducers of apoptosis in BEAS-2B epithelial cells through simultaneous activation of intrinsic and extrinsic pathways. OMVs from hv*Kp* strains containing LPS and OMPs disrupt mitochondrial integrity, promote Cytochrome C release, and activate caspase-9, while also engaging Fas/FasL-mediated caspase-8 signaling. This dual activation accelerates epithelial damage, enhances immune evasion, and promotes respiratory pathogenesis [9,57].

Finally, efficient efferocytosis is essential for immune homeostasis, ensuring the clearance of apoptotic neutrophils and preventing secondary necrosis mediated by gasdermin E (DFNA5). *Kp* disrupts this process by altering flippase activity, reducing phosphatidylserine exposure on dying neutrophils, and impairing macrophage recognition and uptake. This blockade of efferocytosis sustains unresolved inflammation, facilitating bacterial persistence and dissemination [10]. Collectively, these findings establish apoptosis modulation as a central virulence strategy employed by *Kp*.

### 5.3. Necroptosis

Necroptosis is a regulated, caspase-independent form of inflammatory cell death initiated by death receptors (e.g., TNFR1), Toll-like receptors, and intracellular nucleic acid sensors. Activation of receptor-interacting protein kinases RIPK1 and RIPK3 leads to phosphorylation and oligomerization of mixed lineage kinase domain-like protein (MLKL), resulting in membrane rupture and release of pro-inflammatory mediators [11]. While necroptosis can restrict pathogen replication, excessive activation contributes to tissue injury and immunopathology.

**Figure 3:**
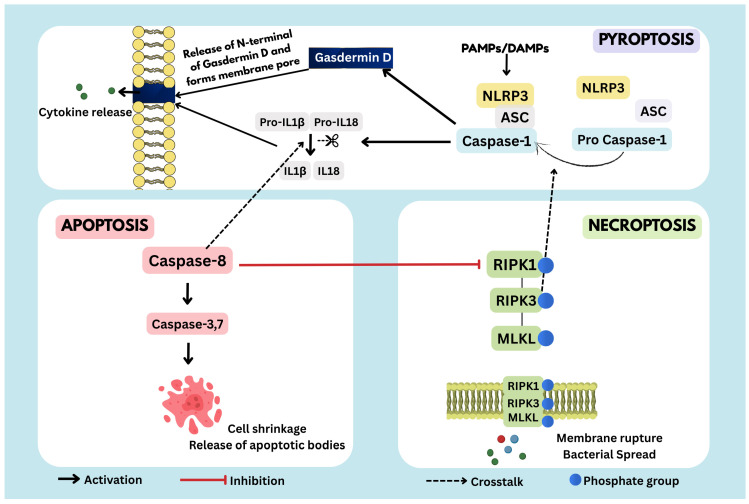
Crosstalk between major cell death pathways. Schematic representation of the interplay between apoptosis, pyroptosis, and necroptosis, highlighting key molecular nodes involved. Caspase-8 serves as a central regulator, activating apoptosis while inhibiting necroptosis. RIPK3 enhances inflammasome activation, whereas caspase-8 induces cytokine release under certain conditions.

RIPK3, a central mediator of necroptosis, also modulates inflammasome activation by promoting caspase-1 cleavage and subsequent maturation of IL-1β and IL-18, thereby linking necroptotic and pyroptotic signaling [58]. Although necroptosis is executed by MLKL and pyroptosis by GSDMD cleavage via caspase-1 or caspase-11, both pathways can be triggered by bacterial components and contribute to inflammatory tissue damage [59]. Notably, both caspase-8 and caspase-1 can process pro–IL-1β, underscoring crosstalk between apoptotic, necroptotic, and pyroptotic signaling cascades [60] which is represented in Figure 3.

*Kp* exploits necroptotic and necrotic pathways to promote dissemination. In hepatocyte-derived HepG2 cells, *Kp* infection shifts cell death from apoptosis to necrosis through caspase-7–mediated PARP inactivation. This transition activates endonuclease G (EndoG), apoptosis-inducing factor (AIF), and DFF40, resulting in DNA fragmentation, ATP depletion, and calpain-2–mediated cytoskeletal breakdown, thereby facilitating bacterial spread [61].

In innate immune cells, *Kp* induces necroptosis to impair host defense mechanisms. Necroptotic death of neutrophils and macrophages disrupts efferocytosis and compromises bacterial clearance. Pharmacological inhibition or genetic deletion of RIPK3 restores efferocytosis and enhances bacterial elimination. Consistently, Ripk3^−/−^ mice exhibit improved lung bacterial clearance and reduced TNF-α levels during *Kp* pneumonia, whereas RIPK1 kinase activity appears dispensable, identifying RIPK3 as a promising therapeutic target [6,62]. *Kp* virulence factors, including LPS and the polysaccharide capsule, activate TLRs and TNFR1, leading to sustained TNF-α signaling and RIPK3-dependent necroptosis. This process compromises epithelial barrier integrity, exacerbates lung injury, and facilitates systemic dissemination. Additionally, KP employs pore-forming toxins and T6SS effectors to further destabilize host cells and amplify necroptotic signaling [12].

The role of necroptosis in CR*Kp* infections is complex. High-risk clones such as ST258 suppress CYLD, a deubiquitinase that negatively regulates necroptosis; nevertheless, necroptotic cell death persists, suggesting the involvement of alternative activation pathways. In THP-1 macrophages, necroptosis is confirmed by the observation that inhibition of MLKL (using necrosulfonamide) significantly reduces cytotoxicity and IL-1β production during CRKP infection [62]. These findings highlight necroptosis as a critical driver of inflammation and pathology in *Kp* infections, particularly in the context of AMR.

As discussed earlier, the dysregulation of these cell death pathways can drive immunopathology. In the context of sepsis, the role of these pathways is mainly stage-dependent. During the early phase of infection, the cell death pathways like pyroptosis and necroptosis facilitate pathogen clearance, whereas on uncontrolled activation, they may amplify the cytokine release leading to tissue injury. As the disease progresses, there will be a shift towards apoptosis, leading to loss of immune cells. In *Kp* infection, this balance is further influenced by virulence factors that could promote disease progression. These observations underscore the importance of temporally regulated cell death responses in determining disease outcomes, as discussed in the following section.

## 6. Host Immune and Inflammatory Responses to *Klebsiella pneumoniae* and Sepsis

The lungs serve dual roles as respiratory and immune organs; however, acute infections such as *Kp* pneumonia disrupt this homeostasis, triggering excessive inflammatory responses that culminate in acute lung injury (ALI) or progress to the more severe acute respiratory distress syndrome (ARDS) [63]. Sepsis is a major predisposing factor for ALI and is associated with high mortality rates ranging from 34 to 60%. It is characterized by extensive neutrophil infiltration, endothelial injury, and profound vascular dysfunction [64].

**Figure 4:**
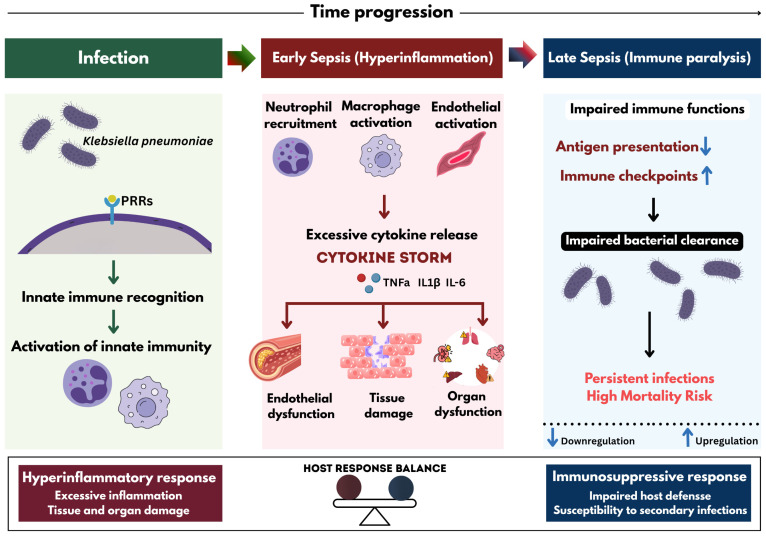
*Kp* infection triggers host immune recognition through pattern recognition receptors, leading to activation of inflammatory signaling pathways and robust cytokine production. The resulting cytokine storm contributes to systemic inflammation, endothelial dysfunction, and tissue injury, ultimately driving the development and progression of sepsis.

Sepsis is a life-threatening organ dysfunction that arises from a dysregulated host immune response. Septic shock represents a more severe subset characterized by circulatory and metabolic abnormalities. Beyond these clinical definitions, sepsis is recognized as a dynamic immunological continuum typically progressing from an early hyperinflammatory phase driven by the excessive production of cytokines and chemokines to a later state of immune suppression. Although these mediators are essential for pathogen clearance, their uncontrolled release leads to widespread inflammation, endothelial dysfunction, tissue injury, and organ failure [18]. This phenomenon, commonly referred to as a cytokine storm, represents an uncontrolled immune amplification loop characterized by excessive cytokine secretion and systemic inflammation. In severe infections, this immune dysregulation can escalate to sepsis, resulting in systemic inflammatory response syndrome (SIRS), cellular damage, multi-organ dysfunction, and potentially fatal outcomes [65] (Figure 4).

Key pro-inflammatory cytokines implicated in sepsis include IL-1β, IL-6, IL-7, and IL-12, which promote immune cell activation, proliferation, and systemic inflammation [65]. In parallel, colony-stimulating factors (CSFs), such as granulocyte–macrophage CSF (GM-CSF), granulocyte CSF (G-CSF), and macrophage CSF (M-CSF), drive emergency myelopoiesis and expansion of myeloid cell populations, further amplifying cytokine production and exacerbating inflammatory pathology [66].

The pathophysiology of sepsis is initiated by innate immune recognition of PAMPs and DAMPs through PRRs [65]. TLR4 serves as a critical sensor of LPS from Gram-negative bacteria, including *Kp*, activating downstream MyD88- and TRIF-dependent signaling pathways. These cascades culminate in the activation of transcription factors such as NF-κB, AP-1, and interferon regulatory factors (IRFs), resulting in the robust inflammatory signaling [15,17].

In addition to TLR signaling, Nod-like receptors (NLRs), particularly NLRP3, play a central role in sepsis-associated inflammation by assembling inflammasome complexes that activate caspase-1. This early phase is marked by the activation of inflammatory cell death pathways, promoting the release of IL-1β and IL-18, thereby intensifying systemic inflammation and immune cell recruitment [17]. Dysregulated inflammatory cell death pathways further compromise tissue integrity and exacerbate organ injury [67].

The TNF–TNFR1 signaling axis is a key regulator of inflammation and cell fate, balancing NF-κB–mediated survival signals with apoptosis or necroptosis, depending on the cellular context (Table 2). RIPK1 plays a critical role in maintaining inflammatory homeostasis by stabilizing TNFR1 signaling complexes. Loss or dysregulation of RIPK1 enhances IL-1α release through NLRP3 inflammasome activation, thereby intensifying inflammatory responses and tissue damage [68]. Endothelial cells actively participate in sepsis pathogenesis by upregulating adhesion molecules such as ICAM-1 and VCAM-1, along with chemokine secretion, which facilitates leukocyte adhesion, transmigration, and tissue infiltration. Concurrently, increased endothelial permeability leads to vascular leakage, edema, and impaired tissue perfusion, ultimately contributing to multi-organ dysfunction [16].

Nosocomial lower respiratory tract infections are a major source of sepsis, especially ventilator-associated pneumonia (VAP) in ICU patients, which significantly elevates sepsis risk [69]. CR*Kp* -induced sepsis causes stronger systemic inflammation, organ dysfunction, and higher mortality than COVID-19 sepsis. *Kp* infection triggers a systemic inflammatory response in humans and mice by activating NF-κB signaling and IRF3 and IRF7 [68]. Dendritic cell activation by *Kp* results in increased IL-12p70 and IL-23 secretion, promoting Th1 and Th17 responses, while failing to induce IL-10, indicating a lack of regulatory immune modulation [19]. Hv*Kp* causes severe cytokine storms through STAT1-mediated immune dysregulation. In murine models, hv*Kp* leads to alveolar macrophage depletion, neutrophil hyperactivation, and M1 macrophage polarization, with significant upregulation of IL-6, IFN-γ, IL-1β, and chemokines Cxcl9 -11, resulting in severe lung damage and mortality within 48 h despite antibiotic therapy [70].

In infected peripheral blood mononuclear cells, lymphocyte proliferation increases in a dose-dependent manner, accompanied by heightened IFN-γ and IL-17A production. Similarly, in A549 pulmonary epithelial cells, *Kp* stimulates IL-6 and IL-8 secretion, further amplifying the inflammatory response [19]. In *Kp* B5055-induced sepsis, an uncontrolled immune response led to persistent inflammation, bacterial overgrowth, and severe ALI, resulting in mortality. Unlike pneumonia, which showed immune balance and resolution, sepsis was marked by elevated IL-1α, TNF-α, NO, and MPO, causing tissue damage. Suppression of IRAK-M expression and absence of alternatively activated macrophages further disrupted immune regulation, exacerbating lung injury [71].

As sepsis progresses, a shift towards immune paralysis occurs, accompanied by a transition from dormant cell death pathways. In *Kp* infection, this imbalance is further exacerbated by bacterial virulence mechanisms that impair effective immune clearance. These stage-specific differences in cell death responses highlight the need for temporally tailored therapeutic strategies, where modulation of inflammatory cell death may be beneficial during early sepsis, whereas preservation of immune cell viability becomes critical in later stages.

## 7. Therapeutic Approaches for Sepsis

Post-mortem analyses of septic patients consistently reveal persistent immune defects, including lymphocyte depletion and impaired antigen presentation, suggesting that immune dysfunction often persists beyond the acute phase. Emerging evidence from small clinical trials indicates that immune-restorative therapies may improve outcomes in selected patients. Similar to oncology, where immune profiling guides therapy, sepsis management may benefit from immunomodulatory strategies tailored to an individual’s immune status. Advances in precision medicine, leveraging genomic, transcriptomic, and proteomic profiling, offer the potential to stratify patients and target specific immune phenotypes, necessitating a paradigm shift from broad, population-based trials to biomarker-guided, individualized interventions [72].

Despite advances in supportive care, most attempts to broadly suppress inflammation in sepsis have failed. Large clinical trials targeting inflammatory mediators demonstrated limited efficacy or unacceptable adverse effects and were ultimately discontinued. The failure of these approaches reflects the complexity of sepsis biology, marked by heterogeneous immune responses, temporal immune shifts, and the absence of reliable biomarkers to guide patient selection. These challenges underscore the need for precision-based immunotherapies rather than indiscriminate immune suppression [72,73].

Recent studies highlight that sepsis is not a uniform immunological state. Some patients exhibit concurrent hyperinflammatory and immunosuppressive features, while others display dominant cytokine suppression. The disease often evolves from early hyperinflammation to late-stage immunoparalysis, with elderly patients being particularly vulnerable due to immunosenescence [74]. Extensive apoptosis of CD4^+^ and CD8^+^ T cells, B cells, and dendritic cells severely compromises host defense, whereas regulatory T cells (Tregs) and myeloid-derived suppressor cells (MDSCs) are relatively preserved, further skewing immune balance toward suppression and increasing susceptibility to secondary infections [75,76]. Strategies that prevent immune cell apoptosis and restore immune competence have been shown to improve survival, emphasizing the therapeutic importance of reversing immune exhaustion rather than solely suppressing inflammation.

The stage-dependent progression of sepsis necessitates specific targeted approaches. During the excessive inflammation phases, pathological activation of inflammatory cell death mechanisms like pyroptosis and necroptosis can exacerbate cytokine release and tissue injury, suggesting that their controlled modulation may limit immunopathology, maintaining host health. In contrast to this, the predominant challenge lies in the excessive loss of immune cells via apoptosis. In this setting, therapeutic strategies should aim to preserve immune cell viability and restore functional competence. Importantly, the significant crosstalk between cell death pathways underscores the need for careful consideration of timing, as interventions that are beneficial during hyperinflammatory conditions may prove detrimental if applied during immune paralysis. Thus, effective therapeutic modulation of cell death in sepsis requires alignment with the evolving immune landscape of the host rather than a uniform treatment approach.

### 7.1. Corticosteroids in Sepsis

The role of corticosteroids in sepsis remains controversial. While large trials have not consistently demonstrated a clear survival benefit, moderate-dose corticosteroids may improve vasopressor responsiveness and limit organ dysfunction in patients with refractory septic shock. Low-dose regimens appear better tolerated and potentially more effective than high-dose approaches. Mechanistically, corticosteroids regulate inflammatory gene expression, inhibit inducible nitric oxide synthase (iNOS), improve endothelial function, and enhance tissue perfusion [77]. In ARDS, corticosteroids suppress NF-κB activation in the lungs and improve cerebral and cardiac perfusion [78]. However, high-dose methylprednisolone has not improved survival and is associated with an increased risk of secondary infections, limiting its clinical utility [79].

### 7.2. Targeted Immunorestorative Therapies

Consistent with the stage-specific nature of sepsis, the immunosuppressive phase is characterized by extensive apoptosis of immune cells, prompting the use of immunorestorative approaches that preserve cell viability and enhance host defense. Targeted immunomodulatory therapies have gained attention for addressing sepsis-induced immunosuppression. GM-CSF enhances monocyte function, restores antigen presentation, and improves immune responsiveness. Clinical studies demonstrate that GM-CSF reduces ICU stay and improves outcomes in septic patients with low monocyte HLA-DR expression [80]. In pediatric sepsis, GM-CSF therapy guided by reduced LPS-stimulated TNF-α production (<200 pg/mL) improved immune function and reduced nosocomial infections, highlighting the value of biomarker-driven treatment [80].

Interleukin-7 (IL-7) is a critical cytokine for T-cell survival and homeostasis. It promotes T-cell proliferation, restores exhausted T cells, enhances trafficking to infection sites, and expands T-cell receptor diversity. In experimental sepsis models, IL-7 improves survival by preventing lymphocyte apoptosis and restoring IFN-γ production. Clinical trials in HIV and cancer patients demonstrate that IL-7 increases CD4^+^ and CD8^+^ T-cell counts without inducing excessive inflammation, supporting its translational potential in sepsis [81,82].

Mesenchymal stem cells (MSCs) represent another promising therapeutic strategy due to their immunomodulatory and regenerative properties. MSCs secrete anti-inflammatory mediators such as IL-10, hepatocyte growth factor, and keratinocyte growth factor, promoting tissue repair and preventing fibrosis, particularly in the lungs. They suppress cytokine storms by inhibiting IL-1, IL-6, IL-12, IFN-γ, and TNF-α signaling, thereby limiting organ damage [83].

In contrast, intravenous immunoglobulin (IVIG) therapy supports bacterial neutralization and opsonization; however, its clinical efficacy in sepsis remains modest, with inconsistent benefits reported across trials [69].

### 7.3. Cytokine-Targeted Therapies

Tumor necrosis factor (TNF), produced by macrophages, T cells, and NK cells, is a central mediator of inflammation, tissue injury, and septic shock [84]. Targeting pro-inflammatory cytokines represents a rational approach for managing cytokine storm syndrome (CSS). Anti-cytokine strategies, including monoclonal antibodies, receptor antagonists, and recombinant proteins, have demonstrated efficacy in inflammatory diseases [68]. TNF neutralization using dimeric TNFR–Fc fusion proteins reduces immune cell infiltration, tissue damage, and mortality in experimental models of bacterial infection and LPS-induced sepsis [85]. AZD9773, a polyclonal ovine Fab fragment targeting TNF, showed mixed clinical efficacy in severe sepsis and septic shock and requires further validation [86]. These therapies may benefit selected patients, particularly those with septic shock, although definitive survival benefits remain uncertain. The clinical success of TNF inhibitors in rheumatoid arthritis underscores their broader therapeutic relevance [87].

IL-1β is a key mediator of excessive inflammation released during pyroptotic cell death. IL-1–targeting therapies, including the monoclonal antibody canakinumab and the IL-1 receptor antagonist anakinra, have demonstrated efficacy in controlling systemic inflammation, including COVID-19–associated ARDS [88,89]. These agents are particularly effective in cryopyrin-associated periodic syndrome (CAPS), caused by NLRP3 mutations leading to uncontrolled IL-1β production [90].

IFN-γ drives immune hyperactivation in hemophagocytic lymphohistiocytosis (HLH) and macrophage activation syndrome (MAS). Neutralization of IFN-γ reduces cytokine-mediated tissue damage and improves survival in preclinical models of HLH, MAS, and endotoxemia-induced sepsis [85]. Emapalumab, an anti–IFN-γ monoclonal antibody, has shown promising results in Phase II/III trials for relapsed or refractory HLH [91,92].

IL-18, released during gasdermin D–mediated pyroptosis, is another potent driver of cytokine storms [93]. Its activity is physiologically regulated by IL-18 binding protein (IL-18BP) [93]. IL-18BP-deficient mice develop MAS-like pathology, which can be reversed by IL-18 receptor blockade. Tadekinig alfa, a recombinant IL-18BP, has demonstrated efficacy in reducing MAS severity in clinical studies [94,95].

### 7.4. Targeting Cell Death and Inflammatory Signaling Pathways

Cytokine-mediated tissue damage in sepsis is closely linked to caspase activation. TNF and IFN-γ activate caspases-3, -7, -8, and -9, driving apoptosis and organ dysfunction. Caspase-8 inhibition reduces macrophage death and improves survival in endotoxemia models [85]. Pan-caspase inhibitors such as zVAD and emricasan have demonstrated survival benefits in sepsis, ischemia–reperfusion injury, and endotoxemia [96]. Broad-spectrum caspase inhibitors (e.g., Z-VAD-FMK, VX-166) reduce apoptosis and inhibit IL-1β and IL-18 release, whereas selective inhibitors like VX-765 suppress caspase-1–mediated pyroptosis [97,98,99]. Nitrosonisoldipine selectively inhibits inflammatory caspases and protects against noncanonical pyroptosis [100]. Although promising, clinical validation remains necessary.

The ALK–EGFR–AKT signaling axis has emerged as a novel therapeutic target. LDK378 (ceritinib), an FDA-approved ALK inhibitor, exhibits potent anti-inflammatory effects and improves survival in lethal endotoxemia models, highlighting its potential repurposing for sepsis therapy [101].

### 7.5. Targeting Pattern Recognition Receptors and Pyroptosis

Therapies targeting PRRs aim to dampen excessive inflammation while preserving antimicrobial immunity. Monoclonal antibodies against TLR4 (e.g., MTS510, 5E3, 1A6) block LPS-induced NF-κB activation and cytokine release, improving survival in murine sepsis models [102,103]. Alternative approaches, including LPS-Trap–Fc fusion proteins and TLR4-BB cell-penetrating peptides, further enhance bacterial clearance while suppressing TLR4 signaling [104,105]. NI-0101, a monoclonal antibody that inhibits TLR4 dimerization, is currently undergoing post-pre-clinical evaluation [106].

TLR2-targeted therapies also show promise. The antagonistic antibody T2.5 blocks Pam3CSK4-induced NF-κB and MAPK activation, improving survival in models of toxic shock and bacterial infection [107]. Combined TLR2 and TLR4 blockade enhances protection against sepsis [103]. Intrabodies such as αT2ib retain TLR2 in the endoplasmic reticulum, reducing TNF production [108]. OPN-305, a humanized monoclonal antibody targeting TLR2/1 and TLR2/6, effectively suppresses cytokine release in inflammatory conditions [109,110].

Several bioactive compounds, including traditional Chinese medicine derivatives (glaucocalyxin A, protopanaxatriol, cinnamon), myricetin, and curcumin analogs, inhibit NLRP3 inflammasome activation and pyroptosis [111,112,113]. Hormonal and metabolic modulators such as estrogen, melatonin, carbon monoxide, and L-epinephrine further regulate pyroptotic pathways, reducing inflammation and organ injury [114,115]. Additional agents—including LL-37, dihydromyricetin, disulfiram, and glutamine—modulate immune responses and show potential in sepsis management. Table 3 represents the clinical status of selected compounds. However, robust clinical validation is still lacking, necessitating further controlled trials [116,117,118].

Despite the promise of such strategies, potential limitations as well as adverse effects must be considered. Most of the cell death pathways exhibit dual roles in host defense and immunopathology. Inflammatory pathways like pyroptosis or necroptosis may contribute to pathogen clearance, and their indiscriminate inhibition may weaken antimicrobial immunity [119]. Similarly, therapeutic suppression of apoptosis to preserve immune cell populations may inadvertently prolong the survival of infected cells, affecting the resolution of infection. Systemic modulation of these pathways may result in off-target effects across multiple tissues. These challenges highlight the importance of carefully balancing immunomodulation with preservation of essential host defense mechanisms [120].

Future therapeutic strategies should integrate mechanistic insights with biomarker-guided patient stratification to enable more precise and effective interventions in sepsis. Molecular signatures associated with specific cell death pathways, including inflammasome components, caspase activation markers, gasdermin fragments, and apoptosis-related factors, may serve as valuable tools for patient stratification and prognostic assessment [121]. Neng Wang et al. reported that C-reactive protein (CRP), procalcitonin (PCT), and IL-6 were the markers commonly used. However, they became obsolete as they were not able to distinguish between sepsis and other inflammatory diseases [122]. Despite the slow progress in the development of sepsis-related biomarkers, continued research is imperative given the urgent clinical demand. A comprehensive and multidisciplinary approach is essential for its development. A hastened regulatory approval board is also very important, considering the need for such treatments for the patients [123]. Integration of such biomarkers with clinical parameters could enable more precise phenotyping of patients. This would potentially help in the selection of appropriate treatment regimens, also based on the disease stage and immune status. Biomarkers are critical cornerstones of clinical decision-making in intensive care unit settings. Its significance is yet to be understood by society, and in the coming years, it should gain attention and potentially reduce the sepsis mortality rate.

**Table 3:** Clinical status and safety profiles from published clinical trials and reviews.

Therapeutic Approach/Compound	Clinical Status	References
Methylprednisolone	FDA-approved drug but adverse effects on immune regulation	[124]
GMCSF	Phase II level	[125]
IL7	Phase II level	[126]
MSCs	Phase II level completed; Further efficacy under investigation	[127]
Intravenous immunoglobulin therapy	As an adjunct therapy; variable efficacies	[128]
AZD9773	Terminated at Phase IIb level due to lack of clinical benefit	[129]
Canakinumab	Not approved for sepsis	[130]
Anakinra	Phase III level completed	[131]
Tadekinig-α	Phase II level	[132]
ZVAD-FMK	Pre-clinical level	[97]
VX166	Pre-clinical level	[98]
VX765	Terminated at Phase IIa level for further clinical development	[133]
Nitrosonisoldipine	Pre-clinical level	[100]
LDK378 (Ceritinib)	FDA-approved oral anticancer drug but yet in pre-clinical level for sepsis	[134]
NI-0101	Phase II for RA but yet pre-clinical for sepsis	[135]
OPN-305	Phase II level	[136]
LL-37 (Cathelicidin)	Phase II trials for foot ulcers but yet pre-clinical for sepsis	[137]
Dihydromyricetin	Pre-clinical level	[117]
Disulfiram	FDA approval for alcohol dependence; pre-clinical in sepsis	[118]
Eritoran	Failed at Phase III level	[138]
TAK-242	Phase II level; further efficacy under investigation	[139]
MCC950	Pre-clinical; Hepatotoxicity halted trials; further efficacy in combination under investigation	[140]

## 8. Conclusions

Pathogens such as *Kp* have evolved sophisticated strategies to subvert host immunity and promote disease progression. This review highlights the multifaceted immune evasion mechanisms employed by *Kp*, including structural modifications of surface components, disruption of innate immune signaling, and targeted manipulation of host cellular responses, all of which collectively enhance bacterial survival and virulence.

A central theme emerging from this review is the strategic modulation of host cell death pathways by *Kp*. Through uncontrolled activation of inflammasomes and caspase-dependent pyroptosis, the pathogen induces excessive inflammation and tissue injury. Simultaneously, *Kp* differentially regulates apoptosis by promoting epithelial cell death to compromise barrier integrity, while inhibiting apoptosis in immune cells to facilitate intracellular survival. In addition, exploitation of necroptotic signaling pathways also disrupts epithelial integrity, enhancing bacterial dissemination. The coordinated regulation of these cell death mechanisms enables *Kp* to exacerbate host pathology. Severe *Kp* infections often progress to systemic immune dysregulation and multi-organ dysfunction. The clinical management of sepsis is increasingly complicated by profound patient heterogeneity, which limits the effectiveness of standardized therapeutic approaches [141].

Host-directed therapies that restore immune balance represent promising alternatives. Over the last few decades, clinical understanding of sepsis and its control strategies has increased. However, the translation into therapeutic strategies is yet to come. Therefore, implementing multifaceted approaches for re-balancing immune dysregulation has to be considered for the development of advanced biomarkers, which could improve the survival of patients. Addressing these knowledge gaps will be pivotal in advancing innovative therapeutic strategies and improving clinical outcomes in *Kp* –associated infections.

## Figures and Tables

**Table 1:** Major genetic variations in the host and its clinical outcome.

Host Gene	Genetic Variation	Mechanistic/Immunological Effects	Clinical Outcome	References
*TLR4*	*Asp299Gly* mutation	Alters LPS recognition and downstream signaling, predisposing carriers to dysregulated inflammatory responses	Increased risk of Gram-negative sepsis	[34,35,36]
*TLR4*	*Thr399Ile* mutation	Hyporesponsive to LPS in culture	Diminished airway response and enhanced susceptibility to sepsis	[36]
*IL10*	*G1082A* SNP	Reduced IL10 expression	Impaired control of inflammation and increased tissue damage	[37]
*IFN-γ*	Polymorphisms in *−1616T*, *+874A*, and *+3234C*	Increased cytokine production	Elevated risks of sepsis initiation	[38]
*TNF-α*	*−308 G→A* promoter polymorphism in *TNF2*	Increased TNF-α production	Excessive systemic inflammatory responses and endothelial dysfunction	[39]
*IL4*	Polymorphisms in IL4	Shift immune responses toward Th2 dominance	Ineffective clearance of intracellular pathogens and increased susceptibility to respiratory infections	[40]

**Table 2:** An overview of receptors and their mechanisms involved during *Klebsiella pneumoniae* infection.

Receptors	Pathway/Mechanism	Effects	References
Toll-like receptors (TLRs)	Recognize LPS, signal through MyD88-dependent and TRIF-dependent pathways, activating NF-κB, AP-1, and IRFs.	Elevates TNF-α, IL-6, IL-1β, and interferons, driving systemic inflammation, endothelial damage, and multi-organ failure in sepsis.	[15,16]
Nod-like receptors (NLRs)	Activation of caspase-1 by the NLRP3 inflammasome promotes the maturation of IL-1β and IL-18.	Amplifies systemic inflammation, drives pyroptosis and necroptosis, worsening tissue damage and immune suppression.	[17,67]
TNF receptor 1 (TNFR1)	Activates NF-κB signaling. RIPK1 stabilizes TNFR1 signaling.	RIPK1 loss activates TNFR1, driving IL-1α release and inflammation via NLRP3. TNFR1 also induces necroptosis, damaging the epithelial barrier and worsening lung injury.	[12,68]
cGAS-STING	Activated by *K. pneumoniae*’s T6SS, resulting in PD-L1 upregulation on macrophages and monocytes.	Suppresses T cell responses, allowing *K. pneumoniae* to evade immune defenses and persist within the host.	[31,32]

## Data Availability

The original contributions presented in this study are included in the article. Further inquiries can be directed to the corresponding author.
